# Radiopacity and physical properties evaluation of infiltrants with Barium and Ytterbium addition

**DOI:** 10.1590/0103-6440202305379

**Published:** 2023-10-27

**Authors:** Priscila Regis Pedreira, Janaina Emanuela Damasceno, Gabriela Alves de Cerqueira, Ana Ferreira Souza, Flávio Henrique Baggio Aguiar, Giselle Maria Marchi

**Affiliations:** 1 Department of Restorative Dentistry, Piracicaba Dental School, University of Campinas, Piracicaba, São Paulo, 13414-903, Brazil.

**Keywords:** Composite Resins, Radiography, Barium, Ytterbium

## Abstract

Radiopaque properties in the infiltrant should be interesting for clinicians to feel more confident to indicate this treatment. Thus, the aim of this study was to evaluate the effect of the incorporation of barium and ytterbium particles on the physical properties of resin infiltrants. Groups were divided according to the addition of ytterbium oxide (Y) alone (30 or 40%) or Y with barium (YB) (15/15% or 20/20% respectively) in the Icon commercial infiltrant and in the experimental infiltrant base. Digital radiography (n=5), Microradiography (n=5), Microtomography (n=3), degree of conversion (n=5), water sorption (n=16), solubility (n=16), contact angle (n=16), flexural strength (n=16), elastic modulus (n=16) and Energy dispersive X-ray Spectroscopy (n=10) were performed. Analyses were performed using the R program, with a significance level of 5%, and microradiography and Microtomography analyses were evaluated qualitatively. In groups with 30 or 40% of ytterbium, radiopacity was higher or equal to enamel. Microradiography and Microtomography appear to have more radiopacity in groups with 40% (Y). Among the groups with no particle addition, those of the experimental infiltrant presented a higher degree of conversion than those of Icon®. In most groups, there was solubility below the ISO-recommended levels. The addition of particles resulted in higher viscosity. Groups with Icon had higher flexural strength and elastic modulus than groups with experimental infiltrant. The addition of 40% (Y) improved polymerization, had low solubility, and had greater radiopacity than enamel, however negatively affected the viscosity increasing then. Experimental groups with the base showed a higher water sorption than Icon groups.

## Resumo

O objetivo desse estudo foi avaliar o efeito da incorporação de partículas de Bário e Itérbio nas propriedades físicas de infiltrantes resinosos. Os grupos foram divididos de acordo com a adição de Itérbio puro (30 ou 40%) ou Itérbio com Bário (15/15% ou 20/20% respectivamente) no infiltrante comercial Icon e no Infiltrante experimental base. Foram realizados os testes de: radiografia digital (n=5), microradiografia Transversa (n=5), microtomografia (n=3), grau de conversão (n=5), sorção (n=16), solubilidade (n=16), ângulo de contato (n=16), resistência flexural (n=16), modulo de elasticidade (n=16) e Espectroscopia por energia dispersiva (n=10). As análises foram realizadas utilizando o programa R, com nível de significância de 5%, e os testes de microradiografia e microtomografia foram analisados qualitativamente. Nos grupos com 30 ou 40% de Itérbio, a radiopacidade foi maior ou igual ao esmalte dentário. Na microradiografia e microtomografia parece ter maior radiopacidade nos grupos com 40% de itérbio. Dentre os grupos sem adição de partículas, os do infiltrante experimental apresentaram maior grau de conversão do que os do Icon e o grupo controle experimental e com 40% de itérbio apresentaram os melhores resultados. Na maioria dos grupos, a solubilidade foi abaixo dos níveis recomendados pela ISO. A adição de particulas resultou em maior viscosidade. Os grupos com Icon apresentaram maior resistência flexural e modulo de elasticidade do que os grupos com infiltrante experimental e a quantidade de partícula aumentou a resistência e o módulo de elasticidade. A adição de 40% de itérbio melhorou a polimerização, apresentou baixa solubilidade e maior radiopacidade do que o esmalte, porém afetou negativamente a viscosidade, aumentando-a.

## Introduction

The concern arises when discussing the treatment of superficial interproximal lesions, given the fact that restorative therapy involves removing a considerable amount of sound tissue, thus bringing the tooth into a circle of treatment and retreatment. ^1^ ^,^ ^2^ Currently, aiming at a minimally invasive and nondestructive odontology, various remineralizing agents could be indicated, such as dental hygiene instruction and fluoride topical application. However, these procedures are limited to the surface of the lesion, not being able to reach the demineralized tissue. ^1^ In addition, requires the cooperation of patients to ensure successful treatment. ^1^ ^,^ ^2^

Some research about infiltration have been done ^3^ ^,^ ^4^ and in 2009 a new micro-invasive treatment method was suggested for the management of white spot lesions (WSL), the resin infiltration technique ^5^. The principle is to use a low-viscosity resin to penetrate the porosities of the demineralized surface through a capillary phenomenon, interrupting the demineralization process and paralyzing the carious lesion. ^1^ ^,^ ^3^ ^,^ ^4^ ^,^ ^5^ Icon® is the unique commercially available resin infiltrant in the market, is mainly composed of light-curable triethylene glycol dimethacrylate (TEGDMA) that allows the infiltration within the WSL by capillarity due to its wettability and viscosity to occlude its pores. ^6^ It is applied mainly to initial white spots, non-cavitated extending radiographically from all enamel to the outer third of dentin on smooth and proximal tooth surfaces. ^6^ ^,^ ^7^

Since Icon was introduced on the market, there have been numerous studies demonstrating their efficacy in preventing and stabilizing the progression of initial proximal caries and the limitations of this material. ^1^ ^,^ ^5^ Infiltrant formulations seeking to improve the only commercial infiltrant on the market have been tested. (6,8-10) Changes have been suggested in order to reduce polymerization shrinkage and/or increase their penetration coefficient, as well as to improve their chemical, physical, and mechanical properties. One of these formulations was studied by Mathias et al. ^8^ based on 25% Bis-EMA, 75% TEGDMA, 0.5% camphorquinone, and 1% ethyl 4-dimethylaminobenzoate (EDAB), with favorable results regarding degree of conversion, sorption, and solubility and was confirmed by Gaglianone et al. ^9^

The commercially available infiltrant does not have radiopacity characteristics, not allowing visualization of penetration depth into the lesions, and thus, the control of the lesion stabilization. ^11^ In addition, the infiltrant should be radiopaque to a certain degree in order to be able to detect secondary caries. ^12^ It is possible to obtain radiopacity either by incorporating radiopaque fillers into polymerizable resins or by using radiopaque monomers. ^12^ Further, radiopacity is not only affected by the amount of filler but also by the type of radiopaque additives used in inorganic fillers. ^13^ Traditionally, radiopacity is provided by using inorganic filler containing high atomic numbers, such as zinc ^30^, strontium (38), yttrium (39), zirconium (40), barium (56), lanthanum (57) and ytterbium (70), which vary greatly in their concentrations. ^12^ ^,^ ^13^

Both barium and ytterbium particles consist of colorless, insoluble crystals in water, are available in white powder form, and are used to give radiopacity in imaging exams. The lower the particle size, the lower the viscosity, and the greater the penetration depth, according to Lee et al. ^14^. Barium oxide is classified as a ground glass powder filler, also known as alkaline glass fillers, with an average size of 0,7μm. ^15^ Whereas Ytterbium (Yb) is a very ductile metal that slowly reacts with water ^16^ with a spherical particle average size of <100 nm. These elements render composites with X-ray radiopacity without the need for additional radiopaque agents, which is beneficial for clinical diagnosis. ^15^ ^,^ ^16^ In addition, Ytterbium is the element with the highest atomic number that is commonly used in composites and Barium is the most common element used in composites to give radiopacity ^15^ ^,^ ^16^.

Therefore, adding filler particles exerting radiopaque properties to the experimental and Icon infiltrant might be a promising enhancement of the infiltration approach, and this should be interesting for clinicians to feel more confident to indicate this treatment for their patients. Thus, the aim of the present *in vitro* study was to evaluate the effect of the incorporation of barium and ytterbium particles on the physical-chemical properties of resin infiltrants. The null hypotheses investigated were that: The addition of radiopaque particles into the infiltrant ^1^ would not confer radiopacity to the material; ^2^ would not compromise viscosity for the penetration depth of the resin infiltrant; and ^3^ would not improve the properties (degree of conversion, water sorption and solubility, contact angle, flexural strength, and elastic modulus) of the modified infiltrants when compared to the control.

## Materials and method

### Infiltrant preparation

In a laboratory, experimental infiltrants were manipulated under yellow light, and controlled humidity and temperature. The particles were incorporated into the Icon infiltrant and into the experimental base using a magnetic stirrer for 24 hours, in the concentrations shown in Box 1, all in percentage by weight. Each experimental infiltrant was stored individually and kept under refrigeration at 4°C. Concentrations of the particles were based on the study of Askar et al. ^7^

Digital radiography (n=5), transverse microradiography (n=5), Microtomography (n=3), degree of conversion (n=5), water sorption (n=16), solubility (n=16), contact angle (n=16), flexural strength (n=16), elastic modulus (n=16) and Energy dispersive X-ray Spectroscopy (n=10) were performed.

### Digital Radiography (DR)

The radiopacity analysis was performed by making a disc-shaped specimen (5mm x 1mm, n = 5) from a silicone matrix. The specimens were photoactivated with an LED light source (Valo) for 40 seconds, and stored at 37ºC for 24 hours. The Kodak Dental Systems digital radiography system (RVG 5000, Eastman Kodak Company, Rochester, NY, USA) was used to perform the analysis. The specimens were positioned together with the film at the center of the sensor, and the aluminum density scale to compare the density. The radiographic apparatus cylinder (Timex 70 E, Gnatus, Osasco, SP, Brazil), 70 kVp and 7mA, was positioned perpendicular to the film, specimen, scale, and tooth at a distance of 4cm of the line until the receptor focus, using an exposure time of 0.05 seconds.

The digital image provided gray values in pixels at the center of each specimen, of each step of the scale, and of the points equidistant from the right and left with a size of 2mm^2^ (44,5X44,5 pixels). This allowed obtaining an average of the radiographic density value. Grayscale comparisons were made to assess and compare the radiopacity level, as evaluated by the histogram contained in the Adobe Photoshop® software. The following equation was used to transform the data to mm:

(A × 0.5) / B + mm to immediately preceding DRM

*A = radiographic density of the material (DRM) - radiographic density of the aluminum increment immediately preceding DRM;*

*B = radiographic density of the aluminum increment immediately after DRM - radiographic density of the aluminum increment immediately preceding DRM;*

*0.5 = 0.5 mm increment of the aluminum scale.*

The density of each specimen was compared with the dentin density (1.23mmAl) and the enamel density (2.24mmAl), represented by the thickness of the aluminum density scale of 1 mm and 2 mm, respectively. Bear in mind that this thickness had to be equivalent to or greater than these respective values, in order to determine the most adequate concentration to distinguish the material.

**Box 1:**
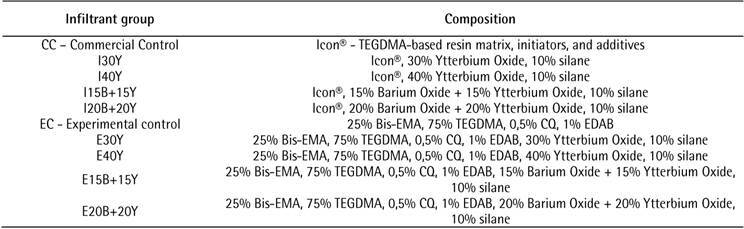
Description of experimental group composition.

### Simulation of caries lesion

### Preparation and selection of test specimens

A total of 50 human molars were used for the experiments, with the approval of the Research Ethics Committee of Piracicaba Dental School - UNICAMP (protocol 41043120.0.0000.5418). From 35 molars, 50 fragments were obtained for the microradiography test, and from the remaining 15 molars, 30 fragments were obtained for the microtomography test. A prophylaxis brush (Microdont, São Paulo, Brazil) and pumice slurry (AAF do Brazil, Londrina, Brazil) were used to remove residues from the teeth, which were then stored in a 0.1% thymol solution. Using a metallographic cutter (Buehler LTD., Lake Bluff, IL, EUA), roots were sectioned from the molars and then discarded. Next, fragments (n =80) were obtained from the enamel portion of the buccal and lingual/palatine faces of 50 teeth, with a dimension of approximately 4mm x 4mm x 2mm. In order to standardize the surfaces, fragments were ground flattened with 600,1200 and 2000 silicon carbide papers (Buehler) under refrigeration, and then polished with felt disks and a diamond solution (1 µm; Buehler) in a polish machine (Arotec S/A Industrial and commerce, Cotia - SP). The fragments were covered with two layers of resistant nail varnish (Colorama®, São Paulo, Brazil), except in the polished enamel area (4 x 4 mm). After, the fragments were stored in *Eppendorfs* with distilled water and taken to an incubator at 37ºC.

### Simulation of the initial enamel caries lesion

A microdurometer (HMV-2000; Shimadzu Corporation, Tokyo, Japan) was used to obtain the initial microhardness average based on three measurements of 100μm from each other, as of the surface center. A simulation of the initial enamel caries lesion was then performed on the fragments. Activity in the oral cavity was simulated using a demineralizing (DE) solution (2.2mmol CaCl2, 2.2 mmol NaH2 PO4, and 50mmol acetic acid, adjusted to pH 4.5 with NaOH) and a remineralization (RE) solution (1.5mmol CaCl2, 0.9mmol P, 130mmol KCl, 0.02mmol buffer solution, adjusted to pH 7.0 with HCl). ^17^ The DE/RE cycle was simulated by immersing the specimens individually into 50mL of DE solution for 6 h, in an incubator at 37°C, washing them with distilled water, and then placed in 32mL of RE solution for 18h and this protocol was performed for 7 days. Two other groups were made to evaluate the correct demineralization of the test specimens: one positive control and one negative control. Following the simulation, a second surface microhardness measurement was performed according to the same parameters described previously with the intention of standardizing the test specimens.

### Infiltration of demineralized enamel

Following the simulation of caries lesion the specimens were randomized, and then infiltrated according to the groups. According to the manufacturer’s instructions (Icon® Etch, DMG, Hamburg, Germany), the enamel was etched with 15% hydrochloric acid for 120 seconds, then washed with a water jet for 15 seconds and dried with air jets for the same time. Icon® Dry was applied for 30 seconds and the infiltrants were applied with a pipette (Microman, Gilson, Middleton, EUA). After a waiting period of 180 seconds for the material to fully penetrate the enamel, a light-curing procedure of 40 seconds was performed using an LED light-curing device (Valo, Ultradent). For all samples in the present study, the infiltrant was reapplied for 60 seconds and light-cured for 40 seconds as recommended by the manufacturer.

### Transverse Microradiography (TMR)

Following the DE/RE cycle and infiltration described before the samples were sectioned perpendicularly to the dental enamel surfaces, and polished to achieve a uniform thickness of 100 µm (± 10) that was verified using a micrometer (Mytutoyo, Japan), n=5. An adhesive tape was used to fix the fragments in a plastic apparatus after they had been prepared. Contact microradiographs of enamel samples were obtained with a nickel-filtered copper (CuKa) X-ray source (PW 1730; Philips, Kassel, Germany) operating at 20 kV and 20 mA on a glass plate. In all microradiographs, an aluminum standard (“step wedge”) with 10 different thicknesses was used to create the control image for gray tone calibration in the software. The distance between the radiation source and the film was 42cm and the exposure time was 13 minutes. After exposure, the glass plate was developed for 4 minutes, fixed for 6 minutes in a dark room at 20^o^C, washed for 10 minutes, and then air-dried. A high-resolution film (FUJIFILM Corporation Japan) was used and developed under standardized conditions according to the manufacturer’s recommendations. The images were digitized by a system (DISKUS; photos and documentation; 4.80 versions; Königswinter, Germany) that is linked to a universal microscope universal (Leica DMRX; Germany).

### Microtomography

Following the DE/RE cycle and infiltration described before the samples (n=3) were attached to the flat end of a brass fitting using wax 7 (Lysanda, São Paulo, Brazil). These apparatuses were exposed to a high-resolution SkyScan 1174 µ-CT SkyScan (SkyScan, Kontich, Belgium) at 50 kV and 800 µA, using a 0.5 mm aluminum filter to eliminate low energy X-rays. Under the program settings, the following parameters were selected: “small camera pixels” (30.04 µm), and rotation steps were adjusted to 0.80º, allowing 360º rotation of the specimen. In a computer, the images are recorded in 2D and 3D for analysis. Each specimen was scanned for 40 minutes in its entirety. 

For image reconstruction, the µ-CT SkyScan software program NRecon (SkyScan, Kontich, Belgium) was used. The software 16-bit angular projection images are stored by the control software and then reconstructs the virtual slices (cross sections). Following reconstruction, the sectional slices were opened in the Dataviewer program (Dataviewer, SkyCan, Kontich, Belgium), which saved coronal, sagittal, and transaxial views, allowing the coronal view to be analyzed. Afterward, all coronal slices of each sample were opened in the CTAn software (SkyScan, Kontich, Belgium), and the region of interest (ROI) for the measurements was selected.

### Degree of conversion (DC)

DC analyses (in%, n=5) were performed by Fourier-Transform Infrared Spectroscopy with attenuated total reflection (FTIR-ATR) (Vertex 70 Spectrometer, Bruker, Billerica, MA, USA). In order to ensure a space of at least 0.2mm, an adhesive tape apparatus was created. The unpolymerized material was first read by depositing approximately 1μL of the infiltrant over the crystal of the device, covering the crystal with a polyester strip for standardization, and focusing the laser beam on the center of the sample. Subsequently, the material was light-cured with an LED light source (Valo, Ultradent, South Jordan, UT, USA, power density of 1000mW/cm^2^, 395-480nm) for 40 seconds, and then a second reading was performed after waiting 2 minutes. To calculate the degree of conversion, the software Opus v.6 (Bruker Optics) was used. Which the corresponding baseline was drawn, and the absorbance value of the baseline was subtracted from the maximum absorbance value at the correspondence wavenumber.

### Water Sorption (WS) and Solubility (SL)

WS and SL tests were performed according to ISO 4049/2009, except on specimen size. Disc-shaped specimens (5mm x 1mm, n=16) were made with the help of a silicone matrix (Scan Putty, Yller) which were then polymerized with an LED light source (Valo) for 40 seconds, placed in a desiccator and stored in an incubator at 37ºC.

An analytical balance (Shimadzu - AUW220D, Tokyo, Japan) was used to weigh the test specimens daily in 24-hour intervals until a constant mass (m1) was obtained, with a variation of less than 0.002mg. In order to calculate each specimen's volume (mm3), the thickness and diameter were measured with a digital caliper (Mitutoyo, Japan). Afterward, the specimens were stored at 37°C in closed Eppendorf tubes containing 1.5 mL of distilled water. The Eppendorf tubes were removed from the incubator after seven days of storage and left at room temperature for 30 minutes. After washing in running water, the specimens were dried gently with absorbent paper, followed by reweighing on an analytical balance to obtain m2. After drying in a desiccator containing silica gel, samples were reweighed daily to obtain the constant mass (m3). The WS and SL values were calculated by two separate formulas (WS = m2-m3 / V and SL = m1-m3 / V).

### Contact Angle (CA)

A goniometer coupled to a camera (Ramé-hart - 500F1, Succasunna, EUA) was used to measure the contact angle (CA) of the infiltrants. Drops (approximately 1μL) were dispensed perpendicular to the surface of a polished glass plate using a disposable syringe (previously covered with insulating tape) attached to a dispersion device and positioned to the equipment. Before use, this polished glass plate was cleaned with absolute alcohol and dried at 37ºC. To obtain the images, the following parameters were used: 10 measurements per sample, an interval of 0.05 seconds, and a frame average of 60 frames/second. Software for measuring angles between the drop and the surface was used to analyze the images (DROPimage Advanced). For each group, the average of the CA on each side of the drop (n=16) was calculated (degrees).

### Flexural Strength (FS) and Elastic Modulus (EM)

In order to evaluate the flexural strength (FS) and the elastic modulus (EM), 16 specimens for each group were made by applying the experimental infiltrants in silicone impressions, made of a bar-shaped matrix (7mm x 2mm x 1mm), defined as a function of the test device. The specimens were photoactivated using a LED (VALO, Ultradent) light source for 40 seconds after a polyester strip had been deposited. Subsequently, the specimens were regularized with silicone carbide papers (1200 and 2000 grit) in a polishing machine with irrigation, and the thickness and height of each specimen were measured with a digital caliper (Mitutoyo, Japan). These dimensions were necessary for the calculation of FS and EM.

Following a period of 24 hours in an incubator at 37ºC, the specimens were subjected to a three-point FS test onan universal testing machine (Instron 4111, Instron Corp, Dayton, OH, EUA) at a speed of 0.5mm/min and under a load of 50N. The maximum load necessary to fracture the specimen as well as its EM were obtained in MPa and GPa, respectively.

### Energy dispersive X-ray Spectroscopy (EDX)

An EDX Link Analytical equipment, model QX 2000, coupled to a Zeiss scanning electron microscope, model LEO 440, was used. After being prepared, sanded, and polished, the samples (n=10) were fixed onto metallic stubs and coated with 30nm carbon. An electron beam of20 keV and a 25mm distance from the gun to the sample were used for analysis. Results are based on an average of five points.

### Statistical Analysis

All analyses were performed with the R* program. First, descriptive, and exploratory analyses of all data were performed. Then, appropriate statistical methods were determined for each variable. Two-way analysis of variance (ANOVA) was used to analyze flexural strength, contact angle, and degree of conversion. Generalized linear models were used for the digital radiopacity, elastic modulus, and sorption variables, as well as considering the effects of infiltrants, particles, and their interactions. The solubility data did not meet the assumptions of parametric analysis and were analyzed by the non-parametric Mann-Whitney tests for the comparisons between infiltrants and Kruskal Wallis and Dunn for the comparisons between particles. In all analyses, a 5% level of significance was considered.

*R Core Team (2021). R: A language and environment for statistical computing. R Foundation for Statistical Computing, Vienna, Austria.

## Results

### Digital Radiography (DR)

There was no significant difference between groups with experimental base and icon in terms of radiopacity (p>0.05). Although, when comparing the addition of particles, radiopacity was significantly higher with 40 Y, followed by 30Y, 20B+20Y, 15B+15Y, and lower in the no-particle group (p<0.05).

### Transverse Microradiography (TMR)

**Figure 1:**
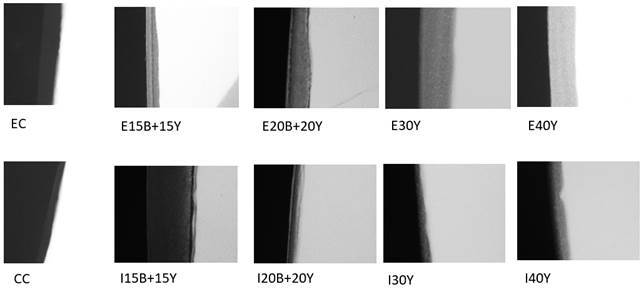
Microradiography of the groups: control commercial (CC), Icon with 15% of barium + 15% of ytterbium (I15B+15Y), Icon with 20% of barium + 20% of ytterbium (I20B+20Y), Icon with 30% of ytterbium (I30Y) and Icon with 40% of ytterbium (I40Y) groups, compared with the aluminum scale; b) shows the radiopacity of specimens of the experimental control (EC), experimental with 15% of barium +15% of ytterbium (E15B+15Y), experimental with 20% of barium + 20% of ytterbium (E20B+20Y), experimental with 30% of ytterbium (E30Y) and experimental with 40% of ytterbium (E40Y).

Figure 1 shows qualitatively the photographic images taken from de microradiographs with the demineralized and infiltrated dental fragment according to the groups: EC, E15B+15Y, E20B+20Y, E30Y, E40Y, CC, I15B+15Y, I20B+20Y, I30Y, I40Y.

 CC and EC showed a dark area (radiolucent) that seems to be a slight enamel demineralization. Moreover, a small level of radiopacity could be seen on the other images, but unfortunately, measurement and conclusion could not be drawn.

### Microtomography

**Figure 2:**
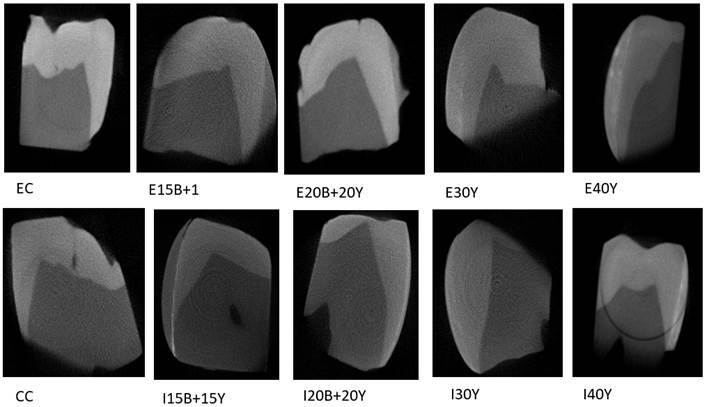
Microtomography of the groups: control commercial (CC), Icon with 15% of barium + 15% of ytterbium (I15B+15Y), Icon with 20% of barium + 20% of ytterbium (I20B+20Y), Icon with 30% of ytterbium (I30Y) and Icon with 40% of ytterbium (I40Y) groups, compared with the aluminum scale; b) shows the radiopacity of specimens of the experimental control (EC), experimental with 15% of barium +15% of ytterbium (E15B+15Y), experimental with 20% of barium + 20% of ytterbium (E20B+20Y), experimental with 30% of ytterbium (E30Y) and experimental with 40% of ytterbium (E40Y) groups.

Figure 2 shows the images taken from the microradiographs with the demineralized and infiltrated dental fragment according to the groups: EC, E15B+15Y, E20B+20Y, E30Y, E40Y, CC, I15B+15Y, I20B+20Y, I30Y, I40Y.

All groups except those without particles exhibit some level of radiopacity, but I40Y appeared to have the highest radiopacity compared to the others.

### Degree of Conversion (DC)

Regardless of the particles, the DC was significantly higher when using the experimental infiltrant than when using Icon (p<0.05), Table 2. For the experimental infiltrant, the highest DC was observed in the groups without particles and with 40Y, while the lowest DC was observed in the 20B+20Y group (p<0.05). As for the Icon infiltrant, the highest DC was observed with the 40Y group, whereas the lowest was seen with the 15B+15Y group (p<0.05).

**Table 2:** Mean and standard deviation of Degree of Conversion, Sorption, Contact Angle, Flexural Strength, and Elastic Modulus (%) as a function of the infiltrant and particles.

Filler concentration	No particle	15B+15Y	30Y	20B+20Y	40Y
Degree of Conversion					
Icon (SD)	49.68 (0.94)Bc	21.44 (1.70)Bd	59.78 (0.22)Bb	48.56 (0.36)Bc	62.41 (0.30)Ba
Experimental (SD)	92.47 (2.25)Aa	52.63 (1.93)Ac	71.59 (1.12)Ab	55.85 (2.55)Ac	91.66 (0.81)Aa
Sorption					
Icon (SD)	59.07 (3.22)Bb	56.47 (3.65)Bc	60.08 (3.08)Bb	53.55 (2.33)Bd	63.73 (4.27)Ba
Experimental (SD)	148.82 (5.13)Aa	120.49 (3.88)Ab	118.85 (4.24)Ab	108.74 (6.29)Ac	107.80(4.82)Ac
Contact Angle					
Icon (SD)	25.04 (2.80)Ad	41.51 (2.86)Ab	36.51 (2.89)Bc	45.61 (2.86)Aa	38.31 (2.54)Bc
Experimental (SD)	20.23 (2.93)Bc	29.87 (2.20)Bb	40.61 (3.26)Aa	31.71 (2.91)Bb	43.33 (2.80)Aa
Flexural Strenght					
Icon (SD)	112.39 (15.89)Aab	113.06 (11.09)Aab	89.51 (11.79)Ac	117.34 (13.44)Aa	102.62 (9.79)Ab
Experimental (SD)	44.41 (5.91)Bd	57.10 (6.11)Bc	69.28 (7.84)Bab	78.39 (9.75)Ba	63.39 (7.46)Bbc
Elastic Modulus					
Icon (SD)	1.78 (0.18)Ab	1.83 (0.23)Ab	1.80 (0.18)Ab	2.11 (0.25)Aa	2.13 (0.22)Aa
Experimental (SD)	0.46 (0.05)Bd	0.68 (0.06)Bc	0.96 (0.15)Bb	0.94 (0.13)Bb	1.15 (0.1)Ba

Different uppercase letters indicate statistical differences in the same column for each test, and different lowercase letters indicate statistical differences in the same line P < 0.05.

### Water Sorption (WS)

Experimental infiltrant WS was higher than Icon under all conditions (p<0.05) (Table 2). For the experimental infiltrant, WS was significantly higher without particles and lower in the 20B+20Y and 40Y groups (p<0.05). For Icon, the 40Y group exhibited significantly higher WS than the others (p<0.05). Moreover, it was also higher in the no-particle and 30Y groups than in 15B+15Y and 20B+20Y (p<0.05). There is even greater WS in Icon at 15B+15Y than at 20B+20Y (p<0.05).

### Solubility (SL)

As for the SL (Table 3), it can be noted that in the conditions 15B+15Y and 20B+20Y, the SL was higher in the experimental infiltrant. In the 30Y and 40Y conditions, it was higher in icon (p<0.05). For the experimental infiltrant, the SL was higher in 15B+15Y and 20B+20Y compared to the other groups (p<0.05). There was a higher SL of Icon in 15B+15Y and 40Y compared to the other groups (p<0.05).

**Table 3:** Mean and standard deviation of solubility as a function of the infiltrant and particles.

Particles	Infiltrant		p-value
	Experimental	Icon	
	Mean (Standard Deviation)	Mean (Standard Deviation)	
No particle	1.74 (2.67) Ab	1.34 (2.39) Ab	0.6511
^1^15B+15Y	17.37 (2.51) Aa	7.52 (2.58) Ba	<0.0001
30Y	-2.26 (2.66) Bb	1.65 (2.20) Ab	0.0018
20B+20Y	19.06 (2.80) Aa	2.49 (2.57) Bb	<0.0001
40Y	1.86 (4.45) Bb	7.5 (3.39) Aa	0.0050
p-value	<0.0001	<0.0001	

^1^B: barium; Y: ytterbium. Different uppercase letters indicate statistical differences in the horizontal and different lowercase letters indicate statistical differences in the vertical (p<0.05).

### Contact Angle (CA)

CA results are shown in Table 2. For the no particles, 15B+15Y, and 20B+20Y groups, CA was significantly lower in the experimental group than in Icon (p<0.05). The experimental infiltrant had a higher CA than Icon in the 30Y and 40Y groups (p<0.05). For the experimental infiltrant, a higher CA was observed with 30Y and 40Y, and a lower CA was observed with no particles (p<0.05). For the Icon infiltrant, a higher angle was observed in the 20B+20Y group and a lower angle in the group without particles (p<0.05).

### Flexural Strength (FS)

FS was significantly higher in Icon than in experimental infiltrant, under all conditions (p<0.05), Table 2. When comparing the experimental infiltrants, 20B+20Y had significantly higher FS than the groups without particles, 15B+15Y, and 40Y (p<0.05). It was also higher at 30Y than 15B+15Y and without particles (p<0.05). For Icon, FS was significantly higher with 20B+20Y than with 30Y and 40 Y (p<0.05). In this infiltrant, the FS was also higher at 40Y than at 30Y (p<0.05).

### Elastic Modulus (EM)

Experimental infiltrants showed lower EM than Icon in all conditions (p<0.05), Table 2. For the experimental infiltrant, EM was significantly higher in the 40Y group and lower in the group without particles than in the other groups (p<0.05). EM was also higher in the 30Y and 20B+20Y groups than in the 15B+15Y and no particles groups (p<0.05). For Icon, EM was significantly higher with 20B+20Y and with 40Y than in the other groups (p<0.05).

### Energy dispersive X-ray Spectroscopy (EDX)

Regardless of the particles, the amount of Ytterbium (Yb) was significantly higher when using the experimental infiltrant than when using Icon (p<0.05), Table 4. For the experimental infiltrant, higher amounts were observed in both 30Y and 40Y groups, and lower amounts were observed in 15B+15Y (p<0.05). As for the Icon infiltrant, the highest amount was observed in the 30Y group and the lowest in the 15B+15Y group (p<0.05).

**Table 4:** Amount of ytterbium (Yb) particles as a function of the infiltrant and particles.

Particles	Infiltrant	
	Experimental	Icon
	Mean (Standard Deviation)	Mean (Standard Deviation)
^1^15B+15Y	61.22 (1.70) Ac	55.31 (1.59) Bd
30Y	91.58 (2.69) Aa	84.31 (5.84) Ba
20B+20Y	67.69 (6.85) Ab	58.51 (2.63) Bc
40Y	96.10 (2.19) Aa	78.75 (7.25) Bb

^1^B: barium; Y: ytterbium. Different uppercase letters indicate statistical differences in the horizontal and different lowercase letters indicate statistical differences in the vertical (p<0.05).

Table 5 shows the results for the amount of Silica (Si). In the 15B+15Y group, there was no significant difference between the infiltrants (p>0.05). In the other groups, the amount of Si was significantly lower in the experimental infiltrant than in the Icon (p<0.05). The amount of Si in the experimental infiltrant was higher in the 15B + 15Y, and it was lower in the 40Y group; in the Icon group, the amount was lower in the 30Y group than in the others (p<0.05).

**Table 5:** Amount of Silica (Si) particles as a function of the infiltrant and particles.

Particles	Infiltrant	
	Experimental	Icon
	Mean (Standard Deviation)	Mean (Standard Deviation)
^1^15B+15Y	22.42 (0.74) Aa	27.88 (1.47) Aa
30Y	8.42 (2.69) Bc	15.69 (5.84) Ab
20B+20Y	16.44 (5.94) Bb	24.96 (2.11) Aa
40Y	3.92 (2.19) Bd	21.26 (7.25) Aa

^1^B: barium; Y: ytterbium. Different uppercase letters indicate statistical differences in the horizontal and different lowercase letters indicate statistical differences in the vertical (p<0.05).

Table 6 shows the results for the amount of barium (Ba). It can be observed that there was no significant difference between the two infiltrants (p>0.05). In both infiltrants, Ba was not detected in groups 30Y and 40Y and there was no significant difference between groups 15B+15Y and 20B+20Y (p>0.05).

**Table 6:** Amount of barium (Ba) particles as a function of the infiltrant and particles.

Particles	Infiltrant		p-value
	Experimental	Icon	
	Mean (Standard Deviation)	Mean (Standard Deviation)	
^1^15B+15Y	16.36 (1.14) Aa	16.81 (1.44) Aa	0.1988
30Y	0.00 (0.00) b	0.00 (0.00) b	-
20B+20Y	15.88 (1.07) Aa	16.53 (1.07) Aa	0.2123
40Y	0.00 (0.00) b	0.00 (0.00) b	-
p-valor	<0.0001	<0.0001	

^1^B: barium; Y: ytterbium. Different uppercase letters indicate statistical differences in the horizontal and different lowercase letters indicate statistical differences in the vertical (p<0.05).

## Discussion

In the current study, experimental resin infiltrants with promising of radiopacity properties were prepared with barium and/or ytterbium oxide with silane. The null hypothesis tested in this study that the addition of radiopaque particles into the infiltrant ^1^ would not confer radiopacity to the material was rejected, since all groups with particles that had radiopacity ^2^ would not compromise viscosity for the penetration depth of the resin infiltrant was rejected, since viscosity increases suggesting that penetration depth could be affected and ^3^ would not improve the properties of the modified infiltrants when compared to the control was accepted, since in most of the tests didn’t improve.

A disadvantage of the infiltrants is their inadequate radiopacity. They are substantially translucent to X-rays and are therefore very difficult or impossible to discern in the event of an X-ray diagnosis. As a result, one of the most important instruments for assessing the extent and location of infiltrations is rendered ineffective. ^13^ Due to the inadequate radiopacity of the infiltrants, it is difficult to determine any caries that might be progressing beneath the infiltrated lesion using X-ray diagnostics, since such caries are very difficult or impossible to distinguish from the infiltrated lesion. ^13^ ^,^ ^18^ Fillers significantly improved the radiopacity of the materials, and their type and content played a critical role in radiopacity. ^13^ The material containing elements of high atomic numbers shows a write shadow due to its high X-ray radiopacity, while the caries with hole structures show black shadow due to the lower X-ray radiopacity. ^18^ Barium has an atomic number of 56, and ytterbium has an atomic number of 70.

There are many factors that affect the radiopacity of dental materials ^13^, but the chemical composition and concentration of the materials seem to be the most important. ^19^ Other factors that can influence the processing system (digital or conventional), exposure settings (time, angulation, speed, and target distance of the Xray film), and material thickness. ^13^ ^,^ ^19^ ^,^ ^20^ Restorative materials should not have a radiopacity that is less than that of enamel, it indicates that they should have a radiopacity similar to or slightly greater than enamel. ^20^

**Figure 3:**
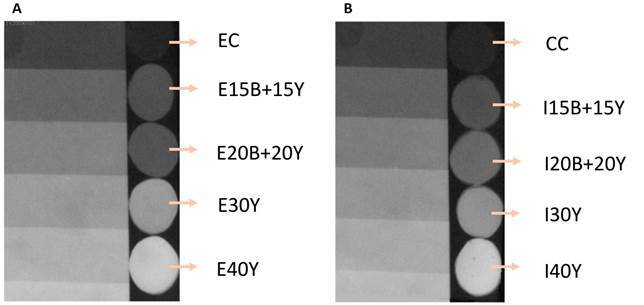
a) shows the digital radiopacity of specimens of the control commercial (CC), Icon with 15% of barium + 15% of ytterbium (I15B+15Y), Icon with 20% of barium + 20% of ytterbium (I20B+20Y), Icon with 30% of ytterbium (I30Y) and Icon with 40% of ytterbium (I40Y) groups, compared with the aluminum scale; b) shows the radiopacity of specimens of the experimental control (EC), experimental with 15% of barium +15% of ytterbium (E15B+15Y), experimental with 20% of barium + 20% of ytterbium (E20B+20Y), experimental with 30% of ytterbium (E30Y) and experimental with 40% of ytterbium (E40Y) groups, compared with the aluminum scale.

The groups with the addition of 30% and 40% of ytterbium obtained higher or equal ​​radiopacity values than those of enamel (2.24 mmAl) (Figure 3). There was a higher radiopacity in the groups with 40% ytterbium, where there was a greater quantity of filler particles. As the filler particle concentration increases in higher atomic number, radiopacity increases as well, as other studies have demonstrated. ^12^ ^,^ ^21^ Mixing ytterbium with barium resulted in lower radiopacity when compared to ytterbium alone in the same proportions (Figure 3), since barium has a lower atomic number, corroborating the findings of previous studies ^21^. A combination of 15% and 20% of barium with 15% or 20% of ytterbium, respectively, was not sufficient to obtain values ​​of radiopacity above that recommended by the International Organization for Standardization (ISO 4049/2009).

The microradiography technique is very sensitive and more indicated to characterize the mineral content of caries lesions, so perhaps it is not ideal for determining the penetration of resin infiltrants. This method requires the preparation of ultrathin samples in order to ensure accuracy of results. ^22^ The demineralized and weakened enamel during sample penetration for the TMR may have broken and it was not possible to observe the lesion and the infiltrant as a whole.

Microtomography images suggest a higher radiopacity when 40% of ytterbium was added which confirms the result in the digital radiography. It is known that the radiopacity of a material increases as the filler particle concentration increases in higher atomic numbers because the absorption capacity of x-rays is increased. ^21^. But unfortunately, this result cannot provide confirmation about the penetration of the infiltrant with particles into the lesion.

Icon is the first ever and so far, the only dental infiltrant, so composition is still unknown, the literature only knows that the main component is TEGDMA (>90%). ^10^ This monomer is hydrophilic, and has a low molecular weight and a high degree of conversion. The degree of conversion may be defined as the consumption of aliphatic double bonds during the curing of a resin. ^7^ For obtain an efficient polymerization and a high degree of conversion depends on a lot of factors i.e.: type of initiation system and concentration of initiators, curing time, sample thickness, filler content, light intensity, chemical structure of monomers and temperature of the atmosphere. ^23^

All groups with Icon had lower DC values than groups with experimental infiltrant base. This result corroborates with previous studies that also found low DC values for Icon ^12^ It can be explained by the excessive amount of TEGDMA in the composition. Despite TEGDMA has a high degree of conversion, dimethacrylate monomer polymerization always leaves a residual amount of unreacted monomer, both because of diffusional limitations and due to the early vitrification the monomer undergoes during polymerization. ^24^ In addition, the presence of BisEMA, a hydrophobic monomer, in experimental groups with infiltrant base may be responsible for the greater polymerization achieved. There is almost no difference in the structure of BisEMA and BisGMA with the exception of the absence of two pendant hydroxyl groups, which leads to lower viscosity and molecular interactions. ^23^ In conjunction with the TEGDMA monomer, which also has low viscosity and no secondary functional groups, a synergic effect results in a greater DC. As a result of these two characteristics, intermolecular interactions become weaker, and segmented diffusion is facilitated, increasing the chance of lattice rearrangement during polymerization. ^25^

The addition of 40% silanized ytterbium to Icon significantly improved the DC and maintained the excellent results obtained in the EC. This fact could be explained due to the resin matrix forms a three-dimensional network structure to encapsulate fillers, after curing. In addition, the coupling agent, silane, facilitates bonding and stress transfer between the filler and the matrix. ^26^

The degradation of resin materials is accelerated by hydrolysis, which reduces their physical-mechanical properties, and, subsequently, their longevity. ^25^ In the present study, high values of WS were found for all groups, since according to ISO 4049/2009, composites may have WS less than or equal to 40μg/mm3. CC presented lower values than EC, this find is not expected since TEGDMA is the main component of Icon (according to the manufacturer). Perhaps, these results are in agreement with those of other investigators ^8^ who also found a lower WS value for Icon. This could be explained because Icon is under patent, therefore, it is unknown how many TEGDMA or other monomers are presented in their composition. However, the solubility of Icon and experimental without particles was low and attempted to the recommended of the ISO 4049/2009 (≤7,5 μg/mm3) as well as when was added 30% or 40% of ytterbium to the infiltrants.

These high values of WS may be related to the polymer chain conformation and weak secondary bonds present in the TEGDMA molecule, as previously described. ^11^ Furthermore, TEGDMA may be released from homopolymers or copolymers, forming a polymer chain prone to chemical degradation. ^10^ Moreover, the silane bond between the organic matrix and the inorganic particles of a composite is susceptible to hydrolysis, which promotes the loss of filler particles from the polymer ^27^, contributing to composite surface alterations. Additionally, unpolymerized monomers, additives, and filler components might be released or leached in water ^18^, thereby also increasing composite degradation. EC and E40y had greater DC and low solubility, since the higher the degree of conversion and thus the lower the amount of unreacted monomer, the lower the solubility value. ^26^

The penetration of a liquid (uncured resin) into a porous solid (enamel lesion) is described physically by the Washburn equation. ^28^ In the case of the infiltrant, the penetration of the liquid is driven by capillary forces. ^29^ Wettability studies usually involve the measurement of contact angles as the primary data, which indicates the degree of wetting when a non-polymerized liquid on a solid surface interacts. The most widely used technique of contact angle measurement of various liquids on polished surfaces is the goniometer. ^7^ ^,^ ^18^ ^,^ ^30^ Small contact angles (<90°) correspond that wetting of the surface is favorable, and the fluid will spread over a large area on the surface, while large contact angles (>90°) generally means that wetting of the surface is unfavorable so the fluid will minimize its contact with the surface and form a compact liquid droplet. ^18^ This is an important property that should be considered for the penetration of resin materials in dental surfaces. ^9^

Kielbassa et al ^29^ noticed that the CA has an effect on the penetrating abilities of resins with a high penetration coefficient into the inner layers of enamel. Dental infiltrants have a low contact angle. A low contact angle indicates hydrophilic properties, which are very desirable in the case of dental infiltrants. ^30^ Infiltrating compounds with low viscosity have limited inner resistance and minimal friction forces within the inner layers of the substance (resin), so they move simultaneously^29^. CA changes depend on the kind of surface topography, surface tension of the liquid, surface energy of the substrate, and level of interaction between the liquid and solid. ^29^ Thus, a glass surface was used instead of dental enamel for the purpose of standardization of the test.

Both EC and CC have lower CA values, with EC showing even lower results. The penetration capacity of resin infiltrant may be due to the low viscosity presented by TEGDMA as well as its low molecular weight, allowing greater penetration of infiltrant compared with other materials, such as sealants and adhesives. ^30^ In addition, the TEGDMA monomer usually exhibits hydrophilic properties because it contains Polyethylene and glycols in the chemical chain. ^30^ Both loading and size (hence the surface area) of the filler particles have a significant effect on the viscosity of the composites. ^31^ In our study, when particles are added the viscosity increases. In experimental groups with base the addition of ytterbium (<100nm) alone had higher CA, this could be explained because of the size of particles when a larger specific surface area resulted from a smaller particle size would lead to more and stronger interactions which in turn lead to the higher viscosity of the composite. ^31^ Since Barium (0,7μm) has a larger particle size than Ytterbium, this may have helped to keep the contact angle from becoming too high. But even with the higher values when particles were added, all groups obtained CA data below 90^o^, which is a desirable factor for infiltrants. ^30^

It is commonly accepted that composites with high DC have good mechanical properties. However, there were several opposing reports in which the DC did not correlate with the mechanical properties of the composite which corroborates with the findings in this study, which the control commercial has a low degree of conversion and high flexural strength and elastic modulus and the experimental control has high degree of conversion and low flexural strength and elastic modulus. ^31^ The optimum filler level for the maximum increase in flexural strength may be affected by the size, shape, and content of the filler particles and the strength of the interactions involved at the filler matrix and filler-filler interfaces. ^14^ ^,^ ^31^ When filler was added in experimental groups with base FS and EM increases. Overall, filler particles give resinous material directly proportional to resistance and viscosity properties. In other words, the higher the amount of filler particles, the better the physical properties of the resin compound, which results in stronger resistance to material deformation and higher viscosity. ^14^

Another aspect worth noting is the fact that fillers often have their surface treated with MPS (methacryloxypropyltrimethoxysilane) in composite materials in order to provide covalent linkage between the more flexible organic matrix to the very stiff inorganic fillers. This link is fundamental to mechanical reinforcement and wear resistance. ^32^ Therefore, particle silanization may have favored the best results.

The EDS analysis is an analytical technique used for the elemental analysis or chemical characterization of a sample ^33^. It was used in this instance to evaluate homogeneity in the solution of the experimental infiltrants. The results could explain that didn’t have a perfect homogeneity with silica and particles among the infiltrants. For example, in barium quantity, they have the same quantity in both mixtures, 15% and 20%. Silica appears to have more quantity in the 30Y than the 40Y.

Although the infiltrants are effective, it is extremely important that Icon has radiopacity since a radiolucent area might be misdiagnosed as a carious lesion if the patient leaves the practice and goes to another dental practice without communicating with the old and new practices. Additionally, proving that the radiolucent resin has been applied to the lesion can be difficult, which may be an issue in a legal dispute. Therefore, the results of the current study are promising. However, further studies are needed to confirm the penetration of both infiltrants and particles into the lesion using other methodologies, since this study had some methodology limitations in confirming the penetration. In addition, maybe the large number of variables tested made it difficult to answer the three hypotheses.

In conclusion, the addition of 40% ytterbium improved polymerization, had low solubility, and had greater radiopacity than enamel, however negatively affected the viscosity increasing then. Experimental groups with the base showed higher water sorption than Icon groups, but all groups obtained higher results than recommended by the ISO.
